# Weakness Due to Anemia? Go Fish! Melena as a Red Herring in the Diagnosis of Statin-Induced Myopathy

**DOI:** 10.7759/cureus.10717

**Published:** 2020-09-29

**Authors:** America S Revere, Benjamin Appelo, Alan Bartholomew, Brandon Kuiper

**Affiliations:** 1 General Surgery, Augusta University Medical College of Georgia, San Antonio, USA; 2 Ophthalmology, San Antonio Uniformed Services Health Education Consortium, Fort Sam Houston, USA; 3 Rheumatology, Brooke Army Medical Center, Fort Sam Houston, USA; 4 Internal Medicine, Brooke Army Medical Center, Fort Sam Houston, USA

**Keywords:** statin, myopathy, weakness, medical error

## Abstract

Statins are a ubiquitous medication class in the primary care setting where they provide effective primary and secondary prevention of coronary artery disease by lowering cholesterol. While statins are mostly safe, muscle-related adverse events are well described. Very rarely patients can actually develop elevated creatine kinase (CK) consistent with myonecrosis. We present a case of progressive anti-hydroxymethylglutaryl coenzyme A reductase (anti-HMGCR) inflammatory myopathy, which was misdiagnosed for many months.

Our patient was a 67-year-old gentleman sent to the ER by the Internal Medicine Clinic for profound weakness and melena. He had recently undergone esophagogastroduodenoscopy (EGD) for evaluation of progressive dysphagia and was found to be significantly anemic. Repeat EGD demonstrated a bleeding ulcer, and his weakness was attributed to anemia; however, careful examination demonstrated objective muscle weakness which could not be attributed to anemia alone. Subsequent work-up demonstrated myositis due to HMGCR antibody. Statin cessation and treatment with steroids and intravenous immunoglobulin (IVIG) led to a nearly full recovery in strength and resolution of dysphagia over the next several months.

## Introduction

Since their introduction in 1987, statins have proven to be an essential and highly effective medication for prevention of coronary artery disease [1]. By inhibiting hydroxymethylglutaryl coenzyme A (HMG-CoA) reductase, an enzyme involved in the rate-limiting step of cholesterol synthesis, statins can lower low density lipoprotein (LDL) cholesterol levels by as much as 60% [2-3]. It is no wonder that atorvastatin and simvastatin are, respectively, the third and fifth most prescribed medications in the United States, with nearly one-fourth of US adults over the age of 40 reporting their use in 2012 [2, 4]. Statins are associated with a few side effects including elevated liver enzymes and myalgia but are generally considered safe. Statin-induced myalgia, in particular, is a frequent complaint, occurring in roughly 2%-20% of patients taking statins [2, 5]. Also known as statin-associated myopathy (SAM), these muscular symptoms occur during treatment and typically resolve upon treatment cessation. The clinical presentation can be highly variable, ranging from muscle tenderness to cramping (myalgia), weakness (myopathy), inflammation (myositis), or muscle breakdown with eventual myoglobinuria or acute renal failure (myonecrosis or rhabdomyolysis) [6-7].

One unique and rare subset of SAM is known as statin-associated autoimmune myopathy (SAAM), with an incidence estimated at two cases per million per year or two of every 100,000 patients taking statins annually [2]. SAAM is uniquely characterized by autoantibodies to HMG-CoA reductase (HMGCR) [2, 7]. Due to its rarity, insidious clinical presentation, and the ubiquitous use of statins, making the diagnosis is often difficult and requires a strong clinical suspicion. We present a case of SAAM that presented to the ER as progressive weakness in the setting of upper gastrointestinal bleed. This case highlights the need for trainees to develop a framework approach to the evaluation of common complaints in order to make the rare diagnosis when clinical presentation deviates from an expected illness script.

## Case presentation

A 67-year-old Hispanic gentleman with past medical history including hypertension, type 2 diabetes, and hyperlipidemia presented to the ED with profound weakness and melena. Although previously active and healthy, he revealed that for the past 8-12 weeks he had been experiencing progressive weakness including difficulty with walking, repeated falls, and dysphagia. He also had a concurrent 30-pound weight loss during this period. As part of a work-up for his dysphagia and weight-loss, he underwent an esophagogastroduodenoscopy (EGD) four days prior which included random gastric biopsies and esophageal dilation due to a small area of stenosis. Afterwards, he reported having five to six episodes of black, tarry stools and worsening of his weakness and fatigue. Notable medications were atorvastatin 40 mg and aspirin 81 mg daily.

Our patient was found to be significantly anemic (hemoglobin, Hgb 8 g/dL, down from 12.8 g/dL measured three weeks prior, normal 13.8-17.2 g/dL ) and to have a hepatocellular pattern of elevated liver associated enzymes (aspartate aminotransferase, AST 272 U/L, alanine aminotransferase, ALT 364 U/L, normal AST 5-40 U/L, normal ALT 7-56 U/L). Repeat labs several hours after admission demonstrated a down-trending Hgb of 7.1 g/dL, which prompted transfusion of two units of packed red blood cells. Gastroenterology was consulted and performed an EGD the morning after admission which demonstrated a few shallow, nonbleeding gastric ulcers thought to have developed from recent biopsy sites.

**Figure 1:**
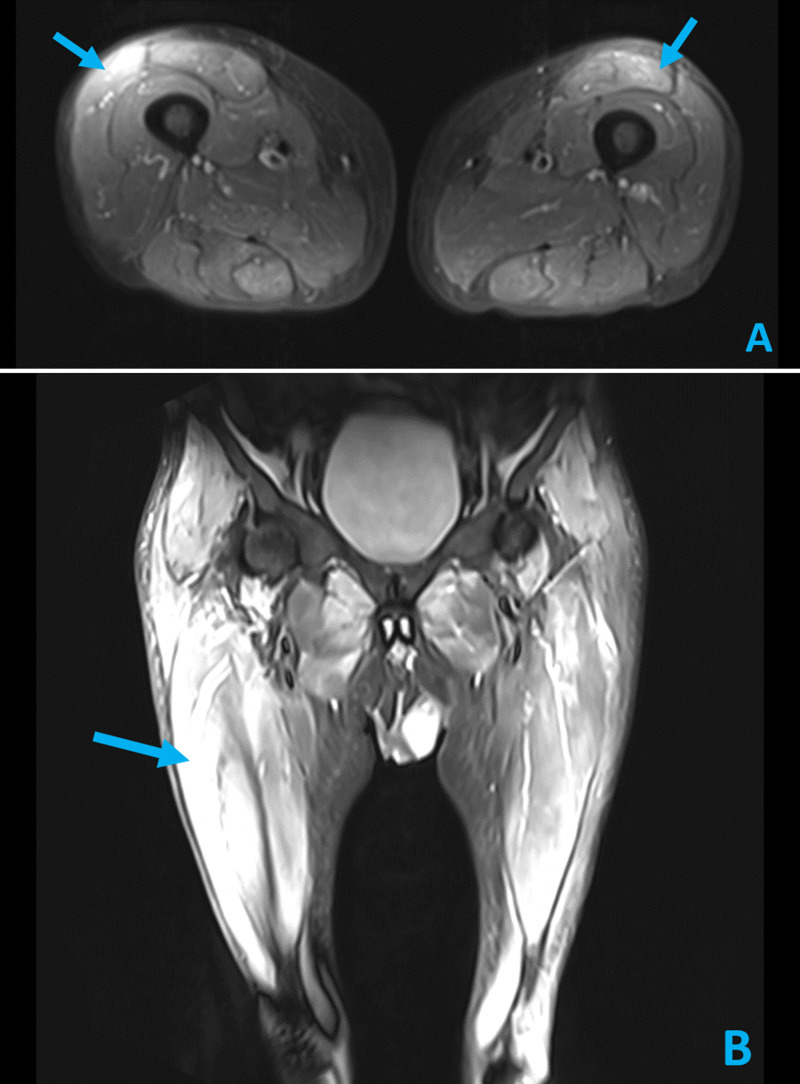
Axial (A) and Coronal (B) STIR MRI images of the lower extremities demonstrating bilateral, multi-compartmental myositis (arrows) with severe involvement of rectus femoris, semimembranosus, and adductor musculature. STIR, Short Tau Inversion Recovery

Though the patient reported less fatigue and mildly improved weakness with his blood transfusion, a careful and thorough physical examination showed only 3/5 bilateral, proximal muscle strength in his upper and lower extremities. In light of objective weakness on exam and reported history of progressive weakness for several weeks, the patient was evaluated for myopathy. With return of labs demonstrating a creatine kinase (CK) of 10,885 IU/L (normal 24-170 IU/L), aldolase >80 U/L (normal 0.5-8.5 U/L), and erythrocyte sedimentation rate (ESR) 60 mm/h (normal 0-22 mm/h), Rheumatology was consulted and a lower extremity MRI was obtained. The MRI found bilateral, symmetric multi-compartmental myositis with severe involvement of the rectus femoris, semimembranosus, and adductor musculature (Figure 1). An HMGCR antibody (anti-HMGCR) and myositis panel were ordered. The patient was started on oral prednisone 60 mg daily and atorvastatin was discontinued empirically.

**Figure 2:**
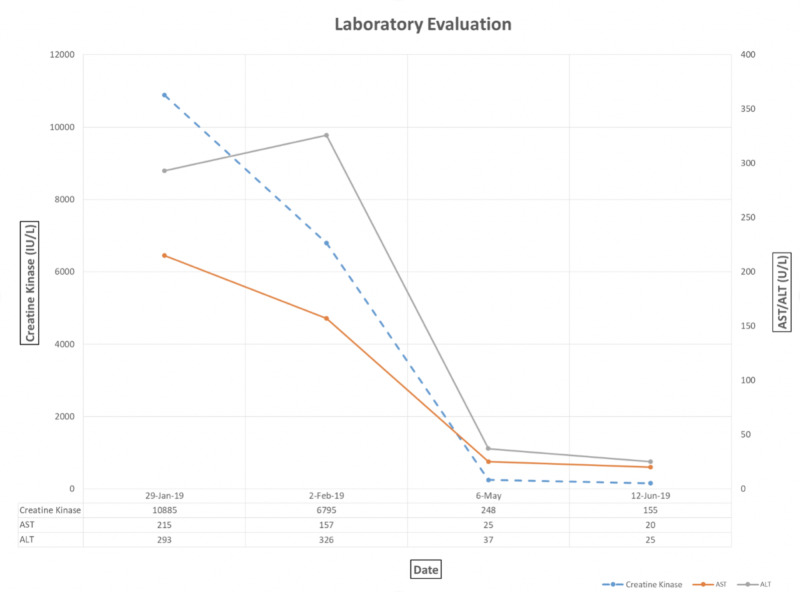
Laboratory evaluation demonstrating marked reduction in CK as well as liver associated enzymes (AST and ALT) over time following induction of high dose daily oral steroid therapy and later monthly IVIG therapy. CK, creatine kinase; AST, aspartate aminotransferase; ALT, alanine aminotransferase; IVIG, intravenous immunoglobulin

Upon discharge, our patient was scheduled for physical therapy and follow-up with rheumatology. A week later, our gentleman was found to be positive for anti-HMGCR and was diagnosed with anti-HMGCR myopathy, related to concurrent statin use. After no significant improvement with oral prednisone alone after one month, intravenous immunoglobulin (IVIG) infusions were added to his treatment plan, which resulted in dramatic improvement of his extremity weakness, resolution of his dysphagia, and marked improvement in serologic markers of muscle breakdown (Figure 2).

Six months after discharge, he reported feeling nearly back to baseline and successfully completed physical therapy. He was slowly tapered off prednisone over a period of 12 months, received six monthly doses of 2 g/kg IVIG infusions, and was transitioned to 2 mg/kg/day azathioprine monotherapy.

## Discussion

This is an interesting case because it both highlights the pathology of a rare diagnosis that presents with a common complaint and exemplifies the critical importance of teaching our trainees to apply a framework approach to diagnosis.

First described in 2010, SAAM is an exceedingly rare diagnosis [2, 7]. The pathogenesis is thought to involve genetically predisposed HLA-types exposed to statins or statin-like natural fungi like certain mushrooms or red yeast rice [8-9]. The statin inhibits the HMG-CoA receptor and upregulates the enzyme expression, triggering an immune response [9]. Muscles are the primary site of necrosis because of the HMG-CoA enzyme presence throughout muscular tissues. This leads to the presenting symptoms, which include bilateral proximal muscle weakness, gait instability, and in extreme cases, dysphagia and diaphragmatic weakness, leading to dyspnea or respiratory failure [9]. Diagnosis is suggested by HMGCR antibodies, CK elevated 10-100 times the upper limit of normal, myopathic pattern electromyography (EMG), and muscle biopsy demonstrating necrosis with prevalent macrophages and minimal inflammation [9]. The MRI characteristically shows diffuse muscle edema. Other common lab findings may include elevated ALT, AST, ESR, C-reactive protein (CRP), and leukocytosis. In contrast to weakness caused by neurological disease, deep tendon reflexes, sensation, and coordination are intact [9].

While cessation of the statin is effective for other statin-associated myopathies (SAMs), cessation alone is not effective for SAAM because of the persistent circulating autoantibodies which make this condition particularly difficult to treat and refractory to many therapies [10]. While no definitive treatment protocol exists, patients are often started on a corticosteroid, but most require additional immunosuppressive or immunomodulating agents, such as azathioprine, mycophenate mofetil, cyclosporine, or IVIGs [9-12]. In recalcitrant cases, plasmapheresis may be used. Demographically, men and women are affected equally by SAAM with an average age of onset of 64 years [2]. Younger patients, with more robust immune systems, typically have a correspondingly more robust immunological response and more severe disease. Notably, the average onset of SAAM occurs 40 months after starting a statin, and there are reports of diagnosis up to 10 years after statin initiation [2]. Fortunately, with appropriate therapy, 91% of patients experience symptom resolution [2].

To prevent severe, progressive disease, which can lead to significant morbidity and death, early diagnosis is essential. In the case of a common chief complaint (i.e., weakness), a systematic approach must be taken to ensure appropriate and timely diagnosis and treatment. Our patient’s prolonged and disjointed evaluation by several providers in different clinics and specialties created some difficulty in the global assessment of the chronicity and severity of his complaints and symptoms; however, each of these stops allowed for an opportunity to take a “diagnostic time-out” to assess his presentation anew [13].

Through a series of presuppositions and anchoring, we can see how our patient’s underlying diagnosis was missed and even led to unnecessary work-up and harm. His earliest complaints of weakness and falls were attributed to deconditioning and it was not until he had developed dysphagia and marked weight loss that he found himself undergoing endoscopic evaluation for suspected gastrointestinal malignancy. It was this EGD with biopsies that directly led to his upper gastrointestinal bleed and anemia that prompted his ER evaluation and admission. Again, his symptoms of weakness were explained away by fatigue in the setting of melena and supposed symptomatic anemia. While symptomatic anemia can cause dyspnea, tachycardia, lightheadedness and fatigue, it does not cause objective muscle weakness on exam. Finally identifying this key piece of evidence led to a careful exam and serological work-up that defined a unifying diagnosis of progressive myopathy. A diagnostic time-out and global assessment of his complaints earlier in his presentation might have spared him an unnecessary procedure and a costly hospital admission.

Demands on resident trainees are significant and compounded by duty-hour restrictions and increased complexity of patient comorbidities, creating an environment where residents have less time to evaluate patients at the bedside and must rely more on “chart biopsy” to extract relevant data to care for their patients [14]. This reality increases reliance on time-saving heuristics and potentiates issues with diagnostic inertia whereby patients are incorrectly labeled with a particular diagnosis which snowballs into further assessment and treatment for that condition, oftentimes overlooking the real etiology and leading to error and morbidity. Some estimates suggest that medical errors are now the third leading cause of patient morbidity in the United States [15]. Diagnostic error is a significant contributor, which emphasizes the value of teaching our trainees to think critically in a systematic fashion using ‘illness-scripts’ and ‘problem representations’ to make accurate diagnostic assessments. As stewards of medical education, it is crucial that clinical educators convey the importance of the diagnostic timeout as a means to reassess the differential diagnosis by promoting a systematic approach to common problems (e.g., anatomic approach to chest pain), emphasizing the importance of the clinical exam, asking “what can’t we explain?,” and embracing the consideration of rare “zebra” diagnoses [13]. Our patient’s gastrointestinal bleed, melena, and anemia were red herrings obscuring the correct underlying diagnosis. A systematic approach generated a complete differential informed by a thorough clinical history and exam that identified a history of progressive weakness that preceded his anemia and objective muscular weakness that could not be explained by anemia alone. This forced further work-up of his condition, led to the unifying but rare diagnosis of SAAM.

## Conclusions

Statin-associated autoimmune myopathy is an extremely rare diagnosis with a nonspecific clinical presentation. If untreated, it can lead to progressive and debilitating weakness with significant patient impairment and decreased quality of life. Unlike other causes of SAM, SAAM requires immunosuppressive therapies in addition to statin cessation. Highlighting similar cases of rare diagnoses with common complaints or uncommon presentations helps strengthen the diagnostic frameworks our trainees use to develop thorough and pointed evaluations that will make them excellent clinicians in the future. 
